# Implication of protein kinase R Gene quantification in hepatitis C Virus Genotype 4 induced Hepatocarcinogenesis

**DOI:** 10.1186/1746-1596-7-103

**Published:** 2012-08-15

**Authors:** Amal A Mohamed, Ola H Nada, Mohamed A El Desouky

**Affiliations:** 1 Department of Biochemistry, National Hepatology and Tropical Medicine Research Institute, Cairo, Egypt; 2 Department of Pathology, Faculty of Medicine, Ain Shams University, Cairo, Egypt; 3 Department of Biochemistry, Faculty of Science, Cairo University, Cairo, Egypt; 4 Ain Shams University-Faculty of medicine-Pathology department-Abbassia, Cairo, Egypt

**Keywords:** Genotype 4 HCV, Hepatocellular carcinoma, Liver, PKR

## Abstract

**Background:**

Protein kinase RNA (PKR-regulated) is a double-stranded RNA activated protein kinase whose expression is induced by interferon. The role of PKR in cell growth regulation is controversial, with some studies supporting a tumour suppressor function and others suggesting a growth-promoting role. However, it is possible that the function of PKR varies with the type of cancer in question.

**Methods:**

We report here a detailed study to evaluate the function of PKR in hepatitis C virus genotype 4 (HCV-4) infected patients. PKR gene was quantitated in HCV related malignant and non-malignant liver tissue by RT-PCR technique and the association of HCV core and PKR was assessed.

**Results:**

If PKR functions as a tumour suppressor in this system, its expression would be higher in chronic hepatitis tissues. On the contrary our study demonstrated the specific association of HCV-4 with PKR expressed in hepatocellular carcinoma (HCC) tissues, leading to an increased gene expression of the kinase in comparison to chronic hepatitis tissues. This calls into question its role as a tumour suppressor and suggests a positive regulatory role of PKR in growth control of liver cancer cells. One limitation of most of other studies is that they measure the levels rather than the quantitation of PKR gene.

**Conclusion:**

The findings suggest that PKR exerts a positive role in cell growth control of HCV-4 related HCC, obtaining a cut-off value for PKR expression in liver tissue provides the first evidence for existence of a viral activator of PKR.

**Virtual Slides:**

The virtual slide(s) for this article can be found here: http://www.diagnosticpathology.diagnomx.eu/vs/1267826959682402.

## Introduction

Chronic infection with hepatitis C virus (HCV) is the predominant aetiology for the development of hepatocellular carcinoma (HCC) worldwide [1-3]. HCV accounts for about 70% of cases with chronic hepatitis, 40% with cirrhosis, 60% with HCC and 15-30% of liver transplantation [4,5]. The prevalence of HCV infection varies throughout the world, the highest number of infections is reported in Egypt [6]. HCV genotype 4 (HCV-4) is common in the Middle East and Africa, where it is responsible for more than 80% of HCV infections. Although HCV-4 is the cause of approximately 20% of the 170 million cases of chronic hepatitis C in the world, it has not been the subject of widespread research [7]. Mechanisms by which HCV infection results in HCC are not well defined, HCV per se increases the risk for HCC through an indirect mechanism mediated by chronic hepatocellular infection; it may also increase the risk of cirrhosis which is by itself a precancerous condition [8]. Hepatocellular carcinoma is the fifth leading cause of cancer death in the world; it is responsible for approximately one million deaths annually worldwide, with a 5 year survival rate of less than 5% [9]. The marked disparity in the incidence of HCC, based on geographic region, always has suggested a role for environment-related and hereditary factors in the development of HCC [10]. Patterns of gene expression in HCC have recently been shown to be of value in predicting prognosis. The genes involved are implicated in cell proliferation and apoptosis [11,12]. Apoptosis is a genetic program of cell death initiated by many different stimuli. Deregulation of the apoptotic process can lead to pathological conditions as cancer, autoimmunity and neurodegeneration [13,14].

PKR (protein kinase RNA-regulated) is a double stranded RNA (dsRNA) activated protein kinase that activates cellular apoptosis pathways [15-17]. PKR is present in a latent or inactive state in cells and is activated by very low concentrations of dsRNAs. Most natural dsRNA activators of PKR are synthesized in virus infected cells as by-products of viral replication or transcription [18,19]. PKR is induced by type I and III interferon, it mediates apoptosis to destroy the cell before the virus can fully replicate and assemble [20,21]. Otherwise, PKR remains inactive and accumulates in the cell leading to continued viral protein translation and viral replication [22]. PKR plays an important role in variety of physiologic processes, including a tumour suppressor function with inhibition of cell proliferation and tumour genesis [23,24]. Increased PKR levels have been observed in a broad range of human tumours but, it is not known whether the loss of PKR activity by inactivating mutations or overexpression of PKR inhibitors in these tumours resulted in higher kinase levels [25-28]. We aim at quantifying PKR gene expression in HCV-4 infected patients and evaluating its role in HCV induced hepatocarcinogenesis.

## Results

### Baseline characteristics

The age of the studied control group GI ranged from 30–73 years with a mean age of 57.24 ±8.87, patients in GII ranged from 30–62 years with a mean age of 45.25 ± 7.84, while GIII ranged from 45–72 years with a mean age of 56.72 ± 6.97. Sex distribution in GI was 12 (60%) females and 8 (40%) males, in GII number of females was 12 (48%) and number of males was 13(52%) while in GIII, there were 7(28%) females and 18(72%) males. There was no statistically significant difference between the three groups as regards the distribution of age and sex.

### Results of laboratory investigation

We found a highly statistically significant elevated median levels of ALT in GII as compared with GI and G III (P < 0.0001and <0.02 respectively) in which the median value of ALT concentration in GI was 29 IU/L with interquartile range (IQR) of 26–35 IU/L, in GII was 41.00 IU/L with (IQR of 37–110 IU/L) while in GIII, it was 40.00 IU/L with (IQR of 30–60 IU/L). Also a highly statistically significant increase in median AST levels was observed in GIII as compared with GI and GII (P < 0.0001) in which the median value of AST concentration in GI was 31 IU/L with (IQR of 26–37.5 IU/L), in GII was 40.00 IU/L with (IQR of 30–60 IU/L) while in GIII, it was 90.00 IU/L with (IQR of 67–140 IU/L). The median value of total bilirubin concentration in GI was 0.8 mg/dl with (IQR of 0.6 -0.9 mg/dl), in GII 1.2 mg/dl with (IQR of 1–1.7 mg/dl) and in GIII 1.1 mg/dl with (IQR of 0.7-2 mg/dl).By statistical analysis there was a very highly significant elevated median levels of total bilirubin in all HCV cases GII and G III as compared with the controls (P < 0.0001 and <0.007 respectively). But there was no statistical significant difference between GII and GIII regarding total bilirubin levels. The median value of serum AFP levels in GI was 6 ng/ml with (IQR of 4–7.25 ng/ml), in GII was 9 ng/ml with (IQR of 6–10 ng/ml) and in GIII was 600 with (IQR of 510–720 ng/ml). A very highly statistically significant elevated median level of AFP was obtained in patients with HCV-4 related HCC as compared with the other two groups (P < 0.0001). The median value of serum viral load in GII was 7000 IU/ml with (IQR of 300-3E + 800 IU/ml) while in GIII median value was 30.000 IU/ml with (IQR of 700-3E + 007 IU/ml). But no significant correlation was detected between serum viral loads in GIII when compared with GII. Genotyping was performed for groups II and III by RFLP method and all cases included in the study were genotype 4.

### Results of liver tissue sample examination

Chronic hepatitis patients showed that 28% had histological activity index score = 0 (no portal inflammation), 44% had score 1–2 (minimal inflammation) and 28% had score 3–8 (mild inflammation). Regarding the stage of fibrosis 64% of patients had fibrosis score 0 (no fibrosis), 12% of patients had score 1 and 24% had score 2. On the other hand, Chronic hepatitis patients with associated HCC showed that 12% of patients had histological activity index (HAI) score 3–8 (mild inflammation), 60% of patients had HAI score 9–12 (moderate inflammation) and 28% of patients had HAI score >12 (severe inflammation). Regarding the stage of fibrosis 40% of patients had fibrosis score 3 (bridging fibrosis), 24% of patients had score 4 (marked bridging fibrosis), and 36% of patients had score 5 (incomplete cirrhosis). The median value of HAI in GII was 2 with (IQR of 0–3) and of fibrosis was 0 with (IQR of 0–1.5) while in GIII, the median value of (HAI) was 11 with (IQR of 9–12) and fibrosis was 4 with (IQR of 3–5). Patients with chronic HCV-4 related HCC (GIII) showed a highly statistically significant higher stage of fibrosis and higher HAI score when compared to patients with chronic hepatitis only (GII), P < 0.0001.

A positive correlation was found between AST and degree of inflammatory activity in which higher AST was associated with higher grades (HAI) of HCV related chronic hepatitis. No correlation was observed between grading and staging of chronic hepatitis and ALT or total bilirubin. There was statistically significant negative correlation between serum viral load and HAI index in both GII and GIII, (p < 0.0001and p = 0.02 respectively) in which the score of HAI increase with the decrease of serum viral load but no statistically significant correlation was detected between fibrosis and serum viral load in GII and GIII. Histopathological examination of HCC cases revealed 6 (24%) well differentiated tumours, 12 (48%) moderately differentiated and 7 (28%) poorly differentiated tumours.

The median value of calculated endogenous reference of PKR gene expression in liver tissue in GI was 24 with (IQR of 23–26) while in GII 23 with (IQR of 2–24) and in GIII, the median value was 211 with (IQR of 28–213). PKR gene expression in liver tissues of GIII was exactly the same in tumorous and non-tumorous tissue. Mann–Whitney test showed that there was a significant elevation in median levels of PKR gene expression in GI (P = 0.023) as compared with GII. Also a very highly significant increase in PKR gene expression was observed in GIII (P < 0.0001) as compared with GII. The results of quantitation of HCV and PKR in both group II and III are shown in (Tables 1 and 2). A significant negative correlation was detected between PKR gene expression in liver tissue and serum viral load in HCV-4 patients and those with HCV-4 related HCC as shown in (Table 3). On the other hand, (Table 4) shows a significant positive correlation between PKR gene expression in liver tissue and both HAI and stage of fibrosis in these studied groups (P < 0.0001 and P < 0.004 respectively). There was no correlation between PKR gene expression in liver tissue and age, ALT, AST, bilirubin, AFP and degree of differentiation in HCC.

**Table 1:** List of PCR results in HCV related chronic liver disease patients (group II)

**Case Number**	**GII (HCV-4)**	
	**Quantitative PKR gene expression**	**Quantitative HCV PCR**
1	2^6^	2x10^1^
2	2^3^	8x10^7^
3	2	6x10^9^
4	2^4^	2x10^3^
5	2^2^	4x10^8^
6	2	4x10^9^
7	2^3^	3x10^6^
8	2^3^	2x10^2^
9	2^6^	3x10^2^
10	2	9x10^8^
11	2	7x10^10^
12	2^2^	4x10^3^
13	2^4^	6x10^3^
14	2^5^	7x10^4^
15	2^8^	2x10^2^
16	2	9x10^2^
17	2^3^	4x10^2^
18	2^6^	3x10^1^
19	2	8x10^9^
20	2	4x10^1^
21	2^5^	7x10^3^
22	2^3^	4x10^6^
23	2^2^	3x10^8^
24	2^5^	4x10^6^
25	2^3^	2x10^2^

**Table 2:** List of PCR results in HCV related HCC patients (group III)

**Case Number**	**GIII (HCV-4 related HCC) same results in tumour and non-tumour tissue**	
	**Quantitative PKR gene expression**	**Quantitative PKR gene expression**
1	2^8^	6x10^8^
2	2^18^	2x10^4^
3	2^9^	7x10^7^
4	2^11^	5x10^8^
5	2^8^	6x10^4^
6	2^7^	4x10^5^
7	2^17^	3x10^4^
8	2^8^	4x10^8^
9	2^15^	3x10^3^
10	2^11^	5x10^6^
11	2^13^	7x10^2^
12	2^9^	5x10^6^
13	2^12^	6x10^7^
14	2^15^	3x10
15	2^11^	7x10^3^
16	2^7^	4x10^8^
17	2^10^	3x10^7^
18	2^13^	4x10^3^
19	2^6^	4x10^2^
20	2^7^	3x10^6^
21	2^9^	7x10^2^
22	2^15^	2x10^2^
23	2^17^	6x10^2^
24	2^11^	3x10^2^
25	2^12^	5x10

**Table 3:** Correlation between hepatitis C virus quantitation in serum and protein Kinase R in liver tissue

	**Serum viral load**	
**PKR gene expression in liver**	**GII** **(HCV-4)**	**GIII** **(HCV-4 related HCC)**
Spearman's correlation coefficient (r_s_)	−0.56	−0.46
***P-value***	0.004	0.02

A significant negative correlation is shown between both studied groups, in which lower serum viral load was associated with higher PKR gene expression

GII (HCV-4): group II with hepatitis C virus genotype 4; GIII (HCV-4 related HCC): group III with hepatitis C virus genotype 4 related hepatocellular carcinoma; PKR: protein kinase R.

**Table 4:** Correlation between grading and staging of chronic hepatitis and protein kinase R in liver tissue

	**PKR gene expression in liver**			
	**GII** **(HCV-4)**		**GIII** **(HCV-4 related HCC)**	
**Spearman's correlation coefficient**	***r***_***s***_	***P-value***	***r***_***s***_	***P-value***
**HAI**	0.83	0.0001	0.89	0.0001
**Fibrosis**	o.56	0.004	0.84	0.0001

A significant positive correlation is obvious in which higher HAI and fibrosis scores are associated with high rate of PKR gene expression.

GII (HCV-4): group II with hepatitis C virus genotype 4; GIII (HCV-4 related HCC): group III with hepatitis C virus genotype 4 related hepatocellular carcinoma; HAI: histological activity.

Index; PKR: protein kinase R.

**Figure 1:**
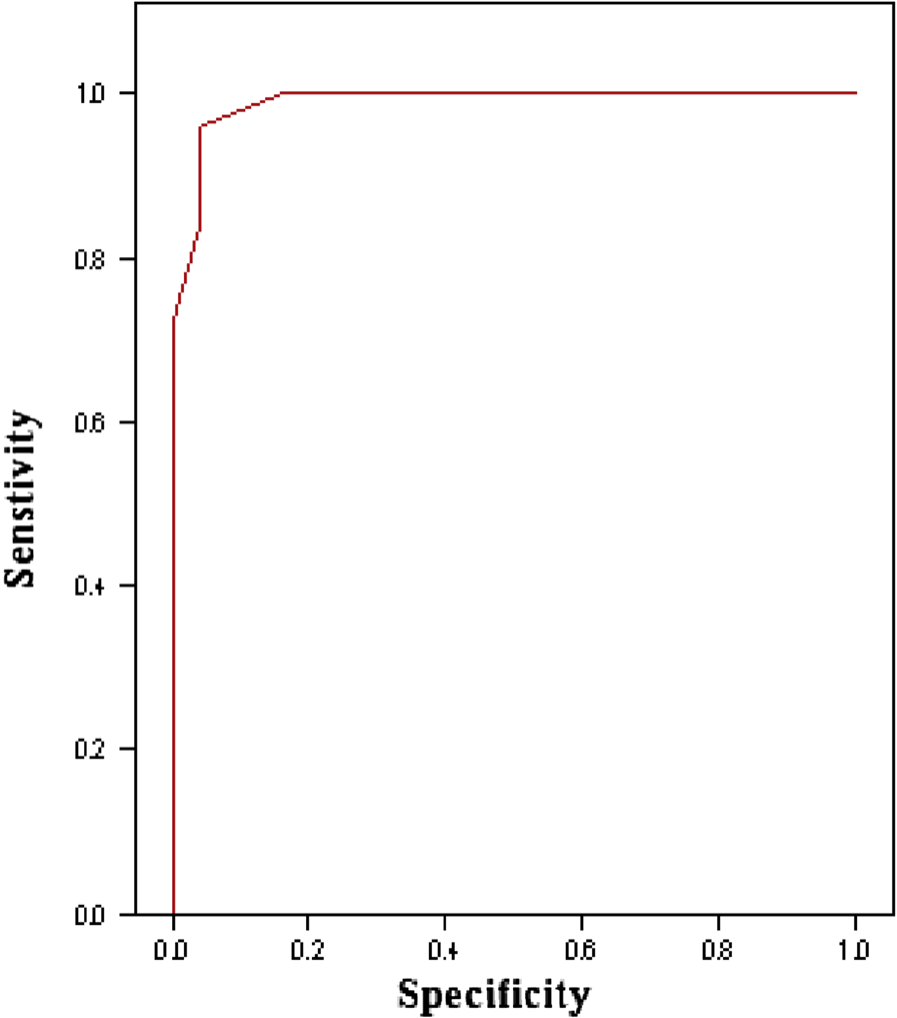
** Sensitivity and specificity of protein kinase R (PKR) quantitation in liver tissue:** Receiver Operating Characteristic (ROC) curve showing highly statistically significant results in which AUC (area under the curve) is 99% and the best cut off value PKR gene expression is 2^7^ with sensitivity of 96%, specificity of 96%, PPV of 96% and NPV of 96% with a diagnostic accuracy of 96% (P<0.0001). Cases with PKR >2^7^ are more likely to be involved with HCC than those with level <2^7^ (odd ratio = 24, 95%CI: 3.5 to 164).

PKR gene quantification was found to be a reliable test to predict HCC development and a cut-off value of 2^7^ was obtained with high sensitivity, specificity and diagnostic accuracy, in which cases with PKR >2^7^ were more likely to be involved with HCC than those with level <2^7^ (P < 0.0001) as shown in (Figure 1).

## Discussion

Chronic infection with HCV is a major health problem affecting more than 170 million individuals in the world. Although the initial infection is largely asymptomatic, prolonged infection carries a high risk of chronic hepatitis, cirrhosis, and HCC. The mechanism of HCV related hepatocarcinogenesis remains to be clarified [5,7]. PKR is an integral part of human innate immune response mechanism, which is the cell's first line of defence against viral infection [24]. The molecular mechanisms that regulate PKR function in normally dividing cells are largely unknown. PKR is implicated in controlling HCV replication and mediating interferon- induced antiviral state against HCV replication [20]. Some viruses including hepatitis viruses can evolve various devices to down-regulate PKR and overcome the host defence mechanism against virus replication [29].

Our results showed that hepatitis C viraemia was much higher in HCC cases but no significance was detected. Others reported a significant increase in viral load after the development of HCC denoting the involvement of HCV in the process of hepatocarcinogenesis [2,3]. On the contrary, Hung observed that HCV replication persists in HCC cells at a lower level than non-malignant cells as they reported significantly lower viraemia in HCC patients [23]. We correlated fibrosis and HAI in all patients with HCV-4 and we got significant results in which patients with chronic HCV-4 related HCC showed higher stage of fibrosis and higher HAI when compared to chronic hepatitis patients without HCC; this may be explained by advanced viral C infection with severe tissue insult. A positive correlation was found between AST but not ALT and the degree of inflammation in chronic hepatitis patients. These results disagree with others who found that ALT was significantly correlated with HAI while, AST was correlated with stage of fibrosis [30]. We obtained a significant negative correlation between viral load and HAI in both HCV-4 chronic hepatitis cases and HCV-4 related HCC cases in which the increase in score of HAI is associated with decrease in the viral load. This correlation was not previously discussed in literature. Consistent with previous reports, no correlation was found between serum HCV-RNA levels and stage of fibrosis in HCV-4 patients [31]. Thus we can deduce that serum HCV-RNA level does not determine the degree of hepatic injury precisely and liver biopsy is mandatory to evaluate the extent of liver damage. In contrast a correlation was suggested by other authors between serum HCV-RNA levels and the fibrosis stage in HCV patients without cirrhosis [32].

HCV participates in the oncogenic mechanisms leading to the development of HCC via inhibiting PKR functions on cell growth control and host apoptotic program [11]. Although, PKR is generally thought to be regulated solely by dsRNA, an intermediate in replication of many viruses including HCV, recent studies have proven the existence of other PKR activation pathways which involve cellular activators that are independent of dsRNA, such as growth factors, lipopolysaccharide, Ca^2+^ and PKR-activating protein [15]. Thus HCV core protein may induce a shift of PKR activity from its classical pathway by targeting other substrates [14,33]. Chen and his colleagues found that PKR level was much reduced in HCC than that in non-tumour tissues [34]. This is not surprising, as PKR is considered a tumour suppressor in some cancer cells. Those cells with low PKR are resistant to apoptosis, thus promoting the growth of tumour. However, in the present study, there was a significantly higher PKR gene expression in HCC tissues and non-tumorous tissues adjacent to malignancy than in tissues of chronic liver disease without a nearby malignancy. Comparing gene expression in chronic liver disease without HCC to those with adjacent malignancy may indicate that higher PKR expression is acquired in premalignant state and during the process of cancer development or even it may be a step in HCV induced hepatocarcinogenesis. This is the first time that PKR-RNA is quantified in HCV-4 related tumour and non-tumour tissues, giving an explanation for the difference from those results obtained by Chen as their study of PKR was immunohistochemical expression, thus lacking a quantitative measurement. Moreover, in our study we have reached a sensitive and specific cut-off for PKR level which can be used in segregation of HCV patients into low risk and high risk groups for tumour development. Some authors support our findings as they suggested that PKR was not acting as a classical tumour suppressor protein but rather as a potential growth stimulus [35]. Also other studies showed that expression of PKR was lower in liver of HCC with HBV than in HCV infection [26,36]. These findings may reflect the difference in viral impact on PKR expression and suggest that PKR might have a tumour promoting action in some cancer cells.

The PKR activation pathway may be inhibited as a survival benefit for the cancer cell. Increased PKR expression has been documented in cancer cells, but its activity was in-fact decreased; therefore we can add that increased PKR expression is a finding but this PKR is non-functioning. This may be attributed to mutation in PKR gene induced by the virus or epigenetics changes whether caused by methylation or phosphorylation. It should however be noted that increased PKR expression was recently described in breast cancer-derived cell lines [25]. Defective programmed cell death mechanisms contribute significantly to the origin and progression of cancer. These tumours show high levels of PKR, suggesting that the ability of PKR to inhibit cell proliferation is impaired in some way [27]. Thus loss of PKR function may constitute one step in the pathway to tumorigenesis. Our observations imply that liver cell proliferation observed during liver carcinogenesis is associated with the selection of viral genomes whose core products activate PKR. The specific association of HCV core protein expressed in HCC tissues with PKR leads to the increased activity of the kinase. PKR activation is not sufficient to cause cell death because of the strong anti-apoptotic signalling in HCV infected cells [16]. Our work showed no significant difference in expression of PKR among patients with well differentiated tumours and those with poor or moderate differentiation, this is in concordance with other study [34]. While a previous report showed that the expression of PKR was increased significantly in well-differentiated HCC related HBV infection, compared with that in poorly differentiated HCC [36]. These different results from ours may be due to the method used for PKR measurement. A TaqMan RT-PCR used in the present study is a quantitative method while the immunohistochemical staining method used in the previous report is not. Also the impact of HCV on PKR may be different from that of HBV.

Further studies showed that normal tissues tend to have lower PKR levels than their neoplastic counterparts [18]. This was in parallel with our data as we found a significant increase in PKR in HCV-4 related HCC cases when compared with normal subjects. There was no correlation between PKR gene expression in liver tissue and age, ALT, AST, bilirubin and AFP. So, PKR was found to be an independent prognostic factor indicating the important biological significance of this gene in the HCC disease process. In a study on peripheral adenocarcinoma of the lung, PKR showed a prognostic impact in which patients with high PKR expression had significantly shorter survival periods than those with low PKR expression [37]. This may be of great help if estimated in HCC patients. We also found that higher PKR gene expression was associated with higher scores of fibrosis and HAI in both HCV-4 and HCV-4 related HCC cases. No other thesis evaluated this relationship.

We detected a significant negative correlation between PKR gene expression and serum viral load in HCV-4 and HCV-4 related HCC cases, in which HCV copy number was significantly decreased by increasing PKR gene expression, these data are compatible with those obtained by others [35]. It appears that HCV protein expression is directly dependent on PKR expression. PKR is antiviral towards HCV thus the over-expression of PKR significantly suppressed HCV levels. From these correlative data, we suggest that PKR is potentially functional in decreasing viral replication. Other authors demonstrated that the expressed endogenous PKR plays a role in controlling the initiation of HCV infection but does not affect HCV replication in infected cells in which persistent viral replication is fully established [5]. Once activated by these low concentrations of dsRNA, PKR cannot be inhibited by subsequent addition of high concentrations of dsRNA [16]. Our findings therefore suggest that the activation of PKR may reduce HCV protein expression and the elimination of infected cells, thus favouring viral persistence.

## Conclusions

We conclude that PKR is involved in the pathogenesis of HCV-4 related HCC by inhibiting viral and cellular proteins related to cell growth and proliferation. PKR gene expression is a reliable marker to predict HCC development and the best cut-off value of PKR gene expression is 2^7^ with high sensitivity, specificity and diagnostic accuracy. We suggest that using a cut-off value of >2^7^ for PKR level might help in identifying HCV patients that are developing HCC. Subsequently, these patients may benefit from novel therapeutic strategies targeting PKR to control malignancy. Further investigations on a larger scale using well-standardized techniques are recommended to validate this cut-off value or define an appropriate one.

## Methods

### Study population

This case control study was conducted on a total number of 70 patients subdivided as follows:

Group I: included 20 normal healthy subjects (as controls to trace changes occurring in the other groups specifically induced by HCV infection), while the cases were represented by Group II: 25 patients with chronic hepatitis C and positive PCR and Group III: 25 patients with proven chronic hepatitis C and developed HCC. Tissue specimens were collected from Ain Shams University Specialized Hospital and Ain Shams University Al-Demerdash Hospital during the period from June 2008 to December 2010.

The Ethical Committee of Ain Shams University Specialized Hospital approved the study protocol, which was prepared in accordance with the ethical guidelines of the 1975 Declaration of Helsinki and later revisions. On admission to the hospital during the study period, every patient fulfilling the inclusion criteria is allocated to one intervention group and informed written consent for the study was obtained from all patients.

The inclusion criteria of HCV cases were: adult male or female (30–73 years old) with proven genotype 4 chronic hepatitis C, positive serum HCV RNA by quantitative polymerase chain reaction (PCR). Those with HCC proved by liver biopsy were allocated to GIII. Besides the necessary investigations needed to fulfill the selection criteria all individuals included in this study were subjected to the following:

1. Full History taking

Full history was taken with special reference to risk factors for HCV infection: as previous exposure to HCV in surgical wards, blood transfusions, dental clinics, needle stick injury, history of HCV in the spouse and i.v. injection.

2. Serum samples

Ten ml of venous blood were withdrawn from each patient in dry sterile vacutainers. After centrifugation, the serum was tested for: a- liver function tests: which included serum aminotransferases (AST), (ALT) and total bilirubin (Beckman Synchron systems; Galway, Ireland). b- Quantitative determination of serum alpha fetoprotein (AFP) level by using Monobind INC. kit USA. c- Quantitation of HCV-RNA using Real Time polymerase chain reaction (RT-PCR) (Strata gene) was done after RNA Extraction (by Viral RNA Extraction Kit, Qiagen-Germany). d- Genotyping of HCV was performed by restriction enzyme digestion (Restriction fragment length polymorphism "RFLP") [38,39]. e- ELISA for HBcAb and HBsAg was done for all cases to exclude presence of hepatitis B viral etiology of liver disease.

3. Tissue Samples

For cases with HCC, liver samples were collected intraoperative after the patients were surgically operated for hepatic lobectomy and two specimens were obtained, one from the tumour tissue and the other from the surrounding non tumour tissue. The Non HCC patients were subjected to ultrasound-guided liver biopsy. All specimens were divided into two portions. The first part was fixed in formalin and embedded in paraffin blocks for histopathological examination. Using a microtome, sections 5 μm thick were cut from formalin-fixed paraffin embedded tissue blocks and subjected for H&E staining and pathological diagnosis, grading and verification of an underlying viral C aetiology. Histopathological grading and staging of chronic hepatitis was done according to Modified Knodell's score [40]. The second part was kept frozen at −80 °C in RNA-Later solution for polymerase chain reaction. Quantification of PKR-RNA gene expression in liver tissue (tumour and non-tumour tissue) was done by RT-PCR using TaqMan® Gene Expression (Applied -Bio systems) after total RNA extraction from liver tissues using Resay Mini Kit (Qiagen Inc, CA Cat-No. 74104). mRNA quantification was calculated by using the arithmetic formula: 2^-Δct.^ In which ΔCT is the difference between the CT of a given target cDNA and an endogenous reference cDNA. Thus, this value yields the amount of the target normalized to an endogenous reference [41].

### Statistical analysis

The data were coded, entered and processed on computer. All statistical work was performed using SPSS (version 15). All values were expressed as mean ± SE. Statistical comparisons were analysed by the two-tailed Student's *t*-test (parametric data) and Mann–Whitney test (non-parametric data). Regression and correlation were done by spearman’s correlation and Pearson's method. A P-value of less than 0.05 was considered the cut-off value for significance.

## Abbreviations

AFP: Alpha fetoprotein; ALT: Alanine aminotransferase; AST: Aspartate aminotransferase; AUROC: Area under receiver operator characteristic; dsRNA: Double stranded ribonucleic acid; HAI: Histological activity index; HBcAb: Hepatitis B core Antibody; HBsAg: Hepatitis B surface antigen; HBV: Hepatitis B virus; HCC: Hepatocellular carcinoma; HCV-4 related HCC: Hepatitis C virus genotype 4 related hepatocellular carcinoma; IQR: Interquartile range; PKR: Protein kinase RNA regulated; RNA: Ribonucleic acid; RT-PCR: Real Time polymerase chain reaction.

## Competing interests

All The authors declare that they have no competing interests.

## Authors’ contributions

AAM: carried out the molecular genetic studies and lab tests. OHN: carried out the histopathological examination and manuscript writing. MAE: participated in study design and coordinated and helped to draft the manuscript. All authors read and approved the final manuscript.
